# Hyperphagia in Prader–Willi syndrome: linking hypothalamic dysfunction to clinical assessment and management across the lifespan

**DOI:** 10.1186/s13023-026-04601-1

**Published:** 2026-10-05

**Authors:** Maithé Tauber, Gwenaëlle Diene, Christine Poitou, Charlotte Höybye, Evelien F. Gevers, Anthony P. Goldstone, Andrea M. Haqq, Yoon Hi Cho, Ashley H. Shoemaker, Janice L. Forster, Anthony Holland, Stephanie Brown, Laura de Graaff, Ann Manzardo, Assumpta Caixàs, Elena G. Bochukova, Paula Maguire, Dairine Dempsey, Jennifer Miller

**Affiliations:** 1 Centre de Référence Maladies Rares PRADORT (syndrome de PRADer-Willi et autres Obésités Rares avec Troubles du comportement alimentaire) and European Reference Network on Rare Endocrine Conditions (Endo-ERN), Hôpital des Enfants, CHU Toulouse, Université Toulouse III, Toulouse, France; 2 Institut Toulousain des Maladies Infectieuses et Inflammatoires (Infinity), INSERM UMR1291-CNRS UMR5051-Université Toulouse III, Toulouse, 31062 France; 3 Sorbonne Université, Assistance Publique–Hôpitaux de Paris, Department of Nutrition and European Reference Network on Rare Endocrine Conditions (Endo-ERN), Pitié-Salpêtrière Hospital, Paris, France; 4 Department of Endocrinology and Department of Molecular Medicine and Surgery and European Reference Network on Rare Endocrine Conditions (Endo-ERN), Karolinska University Hospital and Karolinska Institute, Stockholm, Sweden; 5 Centre for Endocrinology, Queen Mary University of London, Charterhouse Square, London, EC1M 6BQ UK; 6 Department of Paediatric Endocrinology, Royal London Children’s Hospital, Barts Health NHS Trust Royal London Children’s Hospital, Whitechapel, London, E1 1FH UK; 7 PsychoNeuroEndocrinology Research Group, Division of Psychiatry, Department of Brain Sciences, Faculty of Medicine, Imperial College London, Hammersmith Hospital, London, UK; 8 University of Alberta, Alberta, Canada; 9 The Children’s Hospital at Westmead, Sydney, Australia; 10 University of Sydney, Sydney, Australia; 11 Vanderbilt University Medical Center, Nashville, TN USA; 12 Pittsburgh Partnership, Pittsburgh, PA USA; 13 Department of Psychiatry, University of Cambridge, Cambridge, UK; 14 Dutch Center of Reference for Prader-Willi Syndrome and European Reference Network on Rare Endocrine Conditions (Endo-ERN), Rotterdam, The Netherlands; 15 Division of Endocrinology, Center for Adults with Rare Genetic Syndromes, Department of Internal Medicine, Erasmus University Medical Center Rotterdam, Rotterdam, The Netherlands; 16 University of Kansas Medical Center, Kansas City, KS USA; 17 Endocrinology and Nutrition Department, Consorci Corporació Sanitària Parc Taulí. Institut d’Investigació i Innovació Parc Taulí (I3PT-CERCA), Medicine Department Universitat Autònoma de Barcelona, Sabadell, Spain; 18 Blizard Institute, Queen Mary University, London, UK; 19 Soleno Therapeutics Europe, a Neurocrine Biosciences Company, Dublin, Ireland; 20 Division of Endocrinology, Department of Pediatrics, University of Florida, Gainesville, FL USA

**Keywords:** Prader-Willi syndrome, Hyperphagia, Neurobehavioral-metabolic disorder, Neurodevelopmental disorder, Obesity, 15q11-13, Genomic imprinting disorder, Hypothalamic dysfunction

## Abstract

**Background:**

Prader–Willi syndrome (PWS) is a rare genetic neurodevelopmental disorder characterised by hypothalamic dysfunction and profound dysregulation in hunger and appetite regulation, endocrine function, metabolism and behaviour. Hyperphagia is a defining clinical feature and a major contributor to morbidity, mortality and caregiver burden. Increasing evidence suggests that hyperphagia in PWS represents a multidimensional disorder rather than excessive appetite alone.

**Results:**

In December 2024, an international meeting of clinical and scientific experts was convened in Toulouse, France, to explore current understanding of hyperphagia in PWS, challenges in assessment, and evolving approaches to management across the lifespan. Although not a formal consensus process, several consistent themes emerged. These included limitations of existing assessment tools, the need for multidimensional evaluation (including use of instruments such as the Hyperphagia Questionnaire and Hyperphagia Questionnaire for Clinical Trials (HQ-CT). The central role of structured food environments, and vulnerabilities during transition to adult care were also identified as key themes. Experts emphasised that hyperphagia reflects interactions between impaired satiety signalling, activation in reward neurocircuitry, autonomic dysfunction and environmental factors. Management therefore requires integrated biological, behavioural and environmental approaches. While food security and structured support remain foundational, emerging pharmacological therapies may complement multidisciplinary care and reduce caregiver burden.

**Conclusions:**

Hyperphagia in PWS should be conceptualised as a complex, multidimensional disorder requiring comprehensive assessment and integrated management. Future priorities include development of additional validated outcome measures, identification of biomarkers, and care models that better align mechanistic insights with real-world clinical practice.

## Introduction

Prader-Willi syndrome (PWS) is a rare genetic disorder caused by absence of expression of paternally inherited alleles of genes located in the maternally imprinted region of chromosome 15q11-q13 [1, 2]. PWS is increasingly recognised as fundamentally a disorder of brain development in which many of the phenotypic characteristics arise directly from impaired hypothalamic development that affects hunger and appetite regulation, energy balance, endocrine signalling, autonomic function, emotional regulation and behaviour [1, 3]. These multisystem features contribute to a complex phenotype spanning infancy through adulthood with a specific developmental trajectory. PWS has an estimated incidence rate of around 1 in 20,000 live births, although reported prevalence and incidence estimates vary somewhat between populations and healthcare systems, likely reflecting differences in recognition and diagnostic practices. PWS affects males and females equally and has been reported across diverse racial and geographic populations, although ascertainment and diagnosis may vary between settings [1, 3–5]. 

Among the defining clinical characteristics of PWS, hyperphagia is often termed the hallmark feature and in turn is the most clinically consequential [6]. However, the term “hyperphagia” often inadequately captures the lived experience of individuals with PWS and their families. During multidisciplinary discussions, clinicians repeatedly emphasised that hyperphagia in PWS is not simply increased appetite or overeating, but rather a persistent biological drive to seek food, associated with intrusive food-related thoughts, impaired satiety and preoccupation with food often necessitating constant environmental control if severe obesity is to be prevented [7]. While hyperphagia is a core manifestation of the underlying hypothalamic dysfunction in PWS, other behavioural characteristics, including anxiety, rigidity and emotional dysregulation, may influence food-related behaviours and contribute substantially to daily disease burden. In addition to hyperphagia, these associated behavioural challenges can dominate the daily life of both the person with PWS and that of their families and have profound implications, not only for morbidity and mortality but also for independence, education, social participation and family functioning [8–10].

This clinical experience is increasingly supported by advances in biological understanding. Neuroimaging, neuroendocrine and behavioural studies suggest that hyperphagia in PWS arises from disruption of integrated hypothalamic and limbic networks regulating hunger, satiety and reward [11–14]. These findings challenge simplistic behavioural interpretations and support a systems-level model of dysregulated energy homeostasis and food-related behaviour.

Despite these advances in understanding, substantial challenges remain in clinical practice and clinical studies designed to evaluate hyperphagia and underlying causative mechanisms [15]. Assessment of hyperphagia is limited by reliance on subjective measures, while management requires sustained environmental and behavioural strategies that extend beyond conventional care models. Emerging pharmacological therapies offer promise but raise important questions regarding the clinical care setting and integration with established management approaches, in addition to the challenges in assessing effectiveness of novel therapies in this complex condition.

In December 2024, an international expert meeting was convened in Toulouse, France, to explore these issues. Although not designed as a formal consensus exercise, the discussions highlighted recurring themes and unresolved questions across clinical practice and research between leading global PWS experts. This article synthesises these perspectives alongside current evidence, with a focus on linking mechanistic understanding to clinical assessment and management of people with PWS.

## Pathophysiology and clinical phenotype of hyperphagia in PWS

### Hypothalamic dysfunction as a central mechanism

**Fig. 1:**
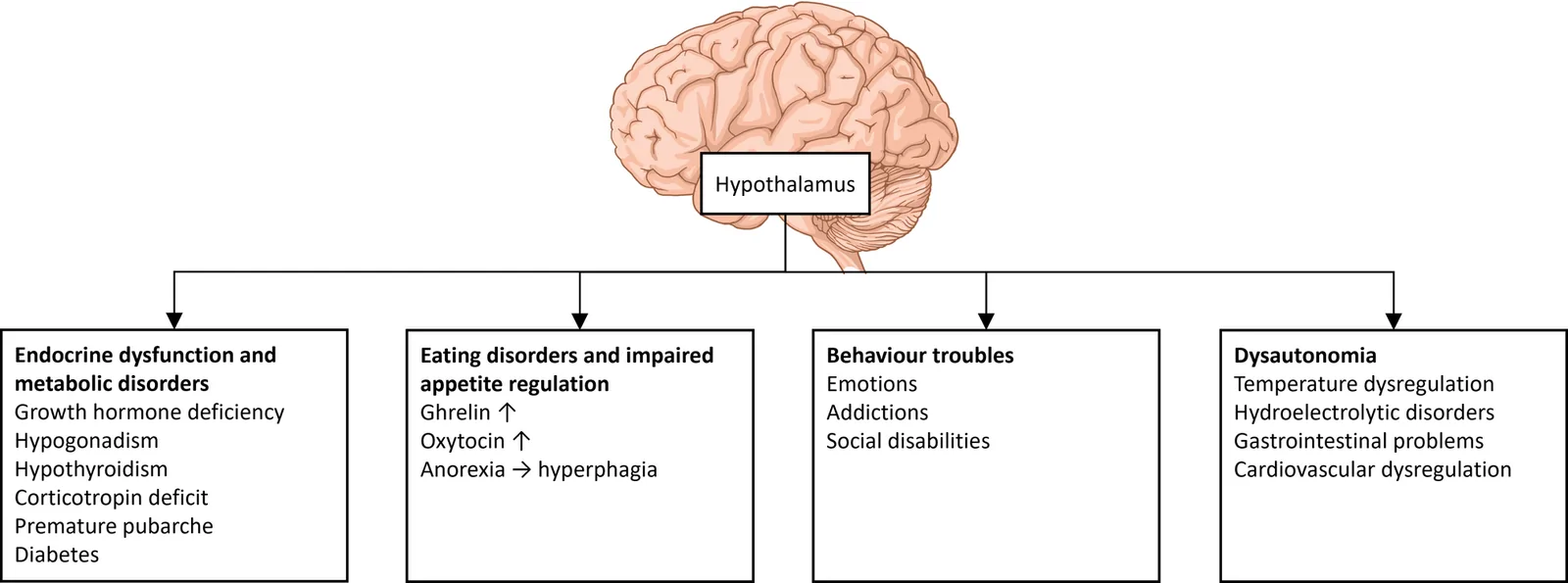
Impaired hypothalamic development and function is the cause of most of the phenotypes. Used with permission from *The Lancet Diab Endocrinol*

The hypothalamus serves as a key integrative centre for regulation of hunger, appetite and energy balance, coordinating peripheral hormonal signals with central neural pathways [16, 17]. In PWS, converging evidence indicates abnormalities in functioning across different components of this system (Fig. 1) [3].

Structural neuroimaging studies have demonstrated reduced hypothalamic volume and alterations across key nuclei involved in hunger and appetite regulation in individuals with PWS [11]. Functional imaging has further shown impaired activation of cortical satiety pathways and exaggerated responses in subcortical regions associated with food motivation and reward [12, 18]. These abnormalities suggest a biological basis for the persistent hunger and impaired satiety observed clinically [12, 19]. 

Participants in expert discussions highlighted how these findings help explain the limited effectiveness of approaches based solely on behavioural control or dietary restriction. Rather than reflecting lack of behavioural restraint, hyperphagia in PWS appears to arise from intrinsic neurobiological dysregulation of hunger and satiety signals. This reframing was considered critical for both clinical management and communication with patients and families.

### Homeostatic dysregulation and endocrine signalling

Neuroendocrine abnormalities in PWS contribute further to dysregulation of appetite and energy balance. Alterations in hormones such as ghrelin, leptin, insulin and adiponectin have been widely reported [20, 21]. 

Elevated circulating ghrelin concentrations, including abnormalities in total acylated and unacylated ghrelin, particularly in fasting states, have been among the most consistent findings [22, 23]. Ghrelin is a potent orexigenic hormone that stimulates appetite and food intake, and its elevation has been hypothesised to contribute to hyperphagia. However, discussions highlighted ongoing uncertainty regarding causality, with some suggesting that ghrelin abnormalities may reflect downstream consequences of hypothalamic dysfunction rather than a primary driver, or a consequence of relatively preserved insulin sensitivity [24]. Burnett et al. proposed one potential mechanism involving abnormal processing of ghrelin precursor to mature ghrelin, possibly linked to reduced prohormone convertase 1 activity. This may be related to altered SNORD116/NHLH2-mediated regulation of PCSK1 [25, 26]. However, hyperghrelinemia has been reported as a distinctive feature of PWS compared with other forms of syndromic obesities, an observation that should be interpreted in the context of differences between total, acylated and unacylated ghrelin, nutritional state and study populations [27]. 

Leptin concentrations in PWS generally correspond to fat mass, suggesting preserved peripheral production, while central leptin responsiveness and downstream hypothalamic signalling may nevertheless be impaired [28, 29]. This again points towards dysfunction at the level of hypothalamic integration.

Endocrinological and neuroimaging findings support the concept that hyperphagia in PWS and associated hypothalamic-related symptoms arise from atypical hypothalamic development and complex interactions across multiple hypothalamic pathways.

### Reward circuitry and the “hungry brain”

Beyond homeostatic mechanisms, increasing attention has focused on altered reward processing and hedonic feeding pathways in PWS. Functional MRI studies showed some overlap between neural activation patterns in PWS and those observed in addiction-related conditions, including increased activation in the amygdala and other reward-associated regions in response to food stimuli [30, 31]. However, what remains unclear is whether increased activation of reward pathways is a normal consequence of the presence of an increased hunger drive or part of the fundamental pathology associated with hyperphagia.

This has led to the concept of a “hungry brain,” in which the persistent food drive is not only a consequence of impaired satiety but also associated with enhanced reward salience and reduced inhibitory control [12, 31]. During discussions, clinicians described patients as existing in a constant state of food vigilance, with food-related cues disproportionately capturing attention and driving behaviour.

At the same time, participants discussed analogies to addiction, particularly in underlying biology and developmental context, as a mechanistic analogy rather than a diagnostic classification or indication that PWS hyperphagia and addiction are equivalent. This neurobiological parallel was considered to provide a useful lens for understanding why environmental control remains necessary to maintain dietary restrictions.

### Developmental trajectory of hyperphagia

Hyperphagia in PWS evolves across well-described nutritional phases [32, 33]. Miller et al. described a gradual developmental progression from phase 0, in utero, through phase 1a, characterised by feeding difficulty, poor weight gain, and failure to thrive (0–9 months); phase 1b, characterised by normal appetite, feeding, and growth (9–25 months); phase 2a, characterised by excessive weight gain without an increase in calorie intake (2.1–4.5 years); phase 2b, characterised by increased appetite and weight gain (4.5-8 years); phase 3, characterised by hyperphagia, food-seeking behaviour, and impaired satiety; and, in some adults, phase 4, characterised by a reduced intensity of hyperphagia [33]. Classic hyperphagia generally corresponds to phase 3, which often begins in mid-childhood, although timing varies substantially between individuals and the transition is not necessarily abrupt.

Discussions highlighted that hyperphagia should not be viewed as a sudden transition but rather a continuum, with subtle behavioural changes often preceding overt food-seeking. Increased focus on food, changes in satiety and early behavioural patterns may signal progression between phases and represent opportunities for early intervention.

Adolescence was repeatedly identified as a critical period. Contributors suggested that worsening hyperphagia during adolescence reflects a convergence of biological and environmental factors, including pubertal neurodevelopment, increasing autonomy, reduced structure and evolving psychiatric vulnerabilities. Based on meeting discussion, adolescence was considered a potential “second inflection point” in clinical management, although this should be viewed as an expert-derived hypothesis requiring further study rather than an established developmental stage.

### Burden beyond appetite

Hyperphagia has profound consequences extending beyond obesity related complications. Caregiver burden emerged as a central theme during discussions, with participants emphasising the constant supervision required to manage food access and prevent harm. Studies have demonstrated strong correlations between hyperphagia severity and caregiver burden, including impacts on mental health, employment and family functioning [34, 35]. Participants highlighted that this burden is often under-recognised in clinical assessments and not adequately captured by traditional outcome measures.

These observations support the view that hyperphagia in PWS is not solely an individual symptom but a condition affecting entire family systems, with implications for how treatment outcomes should be defined and measured.

### Integrating hypothalamic dysfunction with behavioural phenotype

A recurring theme during discussions was the challenge of translating mechanistic insights into clinically meaningful models. While hypothalamic dysfunction is well established in PWS, participants emphasised that the observable phenotype of hyperphagia emerges from the interaction of multiple disrupted systems rather than a single pathway.

In particular, it was noted that classical endocrine models of appetite regulation, centred on discrete hormonal signals such as leptin or ghrelin, do not fully capture the lived phenotype in PWS. Instead, hyperphagia appears to reflect one aspect of broader homeostatic dysregulation arising from hypothalamic dysfunction. Moreover, because emotional regulation is itself impaired in PWS, behavioural problems may in turn be triggered by non-food related stressors, further compounding the clinical picture.

Several expert participants highlighted the importance of considering predictive processing models of brain function as a proposed conceptual framework emerging from the meeting discussion. From this perspective, the brain continuously generates expectations regarding food availability and reward. In PWS, impaired satiety signalling combined with the associated heightened reward salience may lead to persistent mismatch between expected and experienced outcomes, reinforcing food-seeking behaviour. This framework remains theoretical in PWS but may help generate hypotheses regarding why unpredictability around food access can provoke disproportionate distress and behavioural escalation.

These insights reinforce that hyperphagia in PWS is best understood as a systems-level disorder, in which endocrine, neural and behavioural processes are tightly interlinked. This has important implications for both assessment and management, as interventions targeting a single domain are unlikely to fully address the phenotype.

### Heterogeneity in hyperphagia phenotype

Another important discussion point was the variability in hyperphagia severity and presentation across individuals with PWS. While hyperphagia is considered a hallmark feature, its onset, intensity and behavioural manifestations vary considerably. Genetic subtype may contribute to this heterogeneity, with some evidence suggesting differences in neuropsychiatric features between individuals with paternal deletions and those with maternal uniparental disomy [36–38]. These genetic determinants may influence vulnerability to specific behavioural and psychiatric features; however, environmental structure and caregiving context also substantially modify the expression of hyperphagia and food-related behaviours. However, participants noted that genotype alone does not fully explain clinical variability. In 2022, Brown et al. demonstrated that hypothalamic size in PWS was associated with obesity severity and hyperphagia questionnaire for clinical trial (HQ-CT) scores, with smaller hypothalamic size correlated to worse hyperphagia [11]. In addition to these identified biological factors, environmental factors, including family structure, access to specialist care, parents and caregiver’s cultural attitudes towards food and availability of structured support, were considered highly influential. This distinction between biological determinants and environmental modifiers reinforces the importance of viewing hyperphagia as a neurobiological phenotype whose clinical expression is shaped by context.

Although hyperphagia is a defining feature of PWS, it is increasingly recognised as a shared phenotype across a broader spectrum of rare neurodevelopmental disorders (NDDs) associated with genetic obesity. These conditions, including variants affecting the leptin–melanocortin pathway (e.g., MC4R and LEPR deficiencies) and syndromic disorders such as 16p11.2 deletion, Bardet–Biedl syndrome and others, exhibit varying degrees of hyperphagic behaviour, often accompanied by cognitive and behavioural impairments [27, 39–41]. Experts emphasised that increasing knowledge of hyperphagia in PWS will ultimately aid the understanding of hyperphagia in other NDDs.

Understanding this heterogeneity has implications for both clinical management and research design. In particular, it highlights the need for stratification in clinical trials and suggests that treatment response may vary depending on baseline phenotype and environmental conditions.

## Clinical assessment of hyperphagia

### Challenges in assessment

Despite its clinical prominence, hyperphagia is difficult to quantify. Assessment is complicated by developmental variability, environmental control measures and reliance on subjective reporting. A key theme in discussions was that observable behaviour may not accurately reflect underlying severity. In highly structured environments where food access is tightly controlled, individuals may exhibit relatively few overt food-seeking behaviours despite persistent internal drive. As one participant noted, “controlled hyperphagia is still hyperphagia.” This disconnect between internal drive, and observable behaviour presents challenges for both clinical assessment and evaluation of treatment response.

Comparative studies suggest that hyperphagia severity in some NDDs may be comparable to or exceed that observed in PWS, although patterns of onset, behavioural expression and associated comorbidities differ [27, 42, 43]. During expert discussions, participants highlighted the potential value of cross-condition frameworks, including use of standardised tools such as the HQ-CT, to enable comparison across disorders and facilitate phenotype-driven research approaches.

### The challenge of measuring a controlled phenotype

An important conceptual issue raised during discussions is that hyperphagia in PWS is often a “controlled phenotype.” That is, observable behaviours may reflect the success of environmental management rather than the underlying biological drive. In addition, some events of uncontrolled food intake can be totally ignored by caregivers.

This creates a paradox for clinical assessment and research. In highly structured settings, individuals may exhibit relatively few overt food-seeking behaviours, leading to lower hyperphagia questionnaire scores, despite continued intense preoccupation with food. Importantly, this does not necessarily indicate failure of the assessment tool or absence of hyperphagia, as environmental controls may genuinely reduce the behavioural expression of the underlying drive. Conversely, in less structured environments, behaviours may appear more severe, reflecting differences in environmental exposure rather than intrinsic disease severity.

Participants suggested that this distinction between “expressed” and “repressed” hyperphagia may be critical for interpreting clinical data. It also raises important questions regarding how treatment effects should be evaluated. For example, a pharmacological intervention that reduces internal food drive may not lead to large observable behavioural changes in a tightly controlled environment but could still represent meaningful clinical benefit, including improvements in quality of life.

These considerations support the need for assessment frameworks that capture both internal experience and external behaviour. Incorporating caregiver burden, supervision requirements and patient-reported outcomes, with or without connected tools, may help provide a more complete picture of disease impact and need to be evaluated.

### Implications for clinical trials and outcome measures

The limitations of current assessment tools have implications beyond routine clinical practice and extend to clinical trial design. Current instruments to evaluate hyperphagia may not fully distinguish the underlying biological drive from its observable behavioural expression. Participants highlighted a disconnect between endpoints required by regulatory authorities, often focused on changes in HQ-CT scores, and outcomes valued by patients and families, such as reduced anxiety around food, increased independence or decreased need for supervision. In selected circumstances, a reduction in the need for intensive food-related supervision or caregiver monitoring may represent a meaningful clinical benefit, provided this reflects a genuine reduction in hyperphagic drive and can be achieved without compromising safety or metabolic health.

This mismatch may contribute to challenges in demonstrating treatment benefit and may underestimate the long-term real-world impact of interventions. It also underscores the importance of developing outcome measures that better reflect meaningful clinical change. There was considerable interest in exploring composite endpoints that integrate behavioural, functional and caregiver-reported outcomes. While such approaches introduce complexity, they may better capture the multidimensional nature of hyperphagia and provide more relevant information for clinical decision-making. Although instruments such as the HQ and HQ-CT have undergone validation, many proposed multidimensional and composite outcome measures will require further development and validation before widespread implementation.

### Current assessment tools

The original Hyperphagia Questionnaire (HQ) by Dykens et al. and Hyperphagia Questionnaire for Clinical Trials (HQ-CT) are the most widely used instruments for assessing hyperphagia in PWS [44, 45]. These tools capture dimensions of hyperphagic behaviour, drive and severity, and HQ-CT is widely used in clinical trials (Table 1).

**Table 1:** Overview of HQ and HQ-CT

	HQ [44]	HQ-CT [45]
Number of items	11	9
Scoring (diagnostic threshold)	11–55	0–36
Measurement period	1 year	2 weeks
Hyperphagic behaviour (assesses behaviours such as bargaining, foraging, or stealing food)	Range 5–25	Range 0–16
Hyperphagic drive (assesses response when denied food, food-related talk, and persistency in seeking food)	Range 4–20	Range 0–12
Hyperphagic severity (assesses time spent talking about food and how much food interferes with daily routines)	Range 2–10	Range 0–8

HQ, Hyperphagia Questionnaire; HQ-CT, Hyperphagia Questionnaire for Clinical Trials

However, discussions highlighted several limitations. Caregiver-reported measures may underestimate severity when behaviours are suppressed by environmental control. Short recall periods may be influenced by transient factors, and frequency-based scoring may not capture the intensity or consequences of individual episodes.

Other tools, including the Pediatric-Youth Hyperphagia Assessment and measures of behavioural distress or food-related anxiety, may provide complementary information [46–49]. Nonetheless, no single tool provides a comprehensive assessment.

### Toward multidimensional assessment

Participants strongly supported a multidimensional approach to assessment incorporating behavioural observations, including mealtime behaviour, caregiver input, supervision requirements and developmental context.

Repeated longitudinal assessment was considered particularly important, allowing clinicians to detect changes over time and across developmental stages. The importance of clinician experience and multidisciplinary input with therapeutic education sessions of people with PWS and caregivers was also emphasised.

There was also discussion regarding the need for simpler, clinically practical tools for routine use, potentially comprising a small number of core questions capturing key dimensions of hyperphagia that may be electronically filled prior to the visits.

### Biomarkers and objective measures

The potential role of biomarkers generated interest but also caution. Investigational approaches include hormonal markers, food intake behavioural measures, metabolic measures (continuous glucose monitoring in patients presenting with diabetes), eye tracking and neuroimaging [26, 50–52]. 

While these approaches may provide valuable insights into underlying mechanisms and support future research, none are currently validated for routine clinical assessment of hyperphagia in PWS. Participants agreed that they are not currently feasible for routine clinical use. At present, no validated biomarker exists for assessing hyperphagia in PWS in clinical practice. However, biomarkers may ultimately complement clinical assessment and provide objective measures of treatment response, representing an important area for future research.

## Management across the lifespan

### Food security as foundational therapy

A dominant theme of discussions was the central role of food security in managing hyperphagia. Food security refers to predictable, structured control over access to food, including what, when and how much food is available, summarised in the mantra “NO DOUBT, NO HOPE, NO DISAPPOINTMENT” [1].

Participants emphasised that food security should be considered a therapeutic intervention rather than simply environmental management. By ensuring the controlled provision of food, this reduced uncertainty and at the same time eliminated opportunities for unplanned access to food; such structured environments can reduce anxiety and behavioural escalation [49]. 

Importantly, food security addresses not only caloric intake but also the psychological experience of food access. As discussed during the meeting, predictability itself may reduce stress and preoccupation with food.

These approaches must be adapted across the lifespan, with increasing complexity during adolescence and adulthood as autonomy increases.

### Food security as compensation for biological vulnerability

Building on mechanistic insights, participants discussed food security as a compensatory strategy for impaired internal regulation. In individuals without PWS, appetite is regulated by internal signals that guide food intake in response to physiological needs. In PWS, these signals are unreliable or absent, necessitating external structures to fulfil a similar regulatory role.

From this perspective, food security can be conceptualised as an “externalised satiety system,” providing predictable cues that substitute for impaired internal feedback mechanisms. This framing helps explain why strict routines and environmental controls are not merely behavioural strategies but essential components of physiological regulation in PWS.

Participants also emphasised that effective food security requires consistency across environments, including home, school and community settings. Inconsistent expectations and uncertainty around food availability may increase food-related preoccupation and distress and exacerbate behavioural challenges.

### Developmental adaptation of management strategies

Another key theme was the need to adapt management strategies across developmental stages. In early childhood, food security may be implemented primarily through caregiver control and structured routines. As individuals age, increasing autonomy necessitates more complex strategies that balance independence with safety.

Adolescence presents particular challenges, as individuals may seek greater independence while remaining highly vulnerable to environmental cues. Participants noted that failure to adapt management approaches during this period may contribute to clinical deterioration.

In adulthood, additional challenges arise related to housing, employment and social integration. Access to appropriate support structures, including specialist services and residential care where necessary, was identified as a critical determinant of long-term outcomes.

### Transition to adult care

Transition from paediatric to adult services was highlighted as a particularly vulnerable period. Loss of structured support systems, fragmentation of care and increased independence and the associated increase in uncertainty, combined with family exhaustion may lead to worsening hyperphagia and associated complications. This may also be a time when more serious mental health difficulties may emerge for the first time.

Participants emphasised that transition should be viewed not simply as transfer of care but as a critical period requiring active management of both clinical and environmental factors. Published transition frameworks in PWS emphasise that successful transitions to supported accommodation require proactive coordination between specialist healthcare teams, caregivers and residential providers to maintain continuity of specialist care, ensure appropriate food security measures, and minimise the risk of deterioration in metabolic, behavioural and mental health outcomes [53, 54]. Maintaining continuity of expertise and support during this period is essential to supporting quality of life and reducing potential for clinical complications as those with PWS progress through to adulthood [53]. 

### Multidisciplinary and specialist care

Effective management of hyperphagia requires coordinated multidisciplinary care by PWS expert centres involving endocrinology, dietetics, behavioural support, education systems, social care, legal/protection frameworks and adapted residential care homes.

Specialist residential care models have been described as examples of environments where structured food management and multidisciplinary support can be implemented. Observational studies suggest that such settings may be associated with improvements in metabolic and behavioural outcomes; however, these findings should be interpreted cautiously given the potential for selection bias and the limited availability of controlled studies [55, 56]. Adults with PWS often present with complex medical, behavioural and psychosocial needs requiring ongoing specialist input, reinforcing the importance of continuity of multidisciplinary care and access to appropriately structured residential and community support services [57]. 

### Pharmacological therapies

Advances in understanding of the dysregulated hypothalamic and metabolic pathways resulting in hyperphagia have led to the development of targeted pharmacological therapies (Table 2).

**Table 2:** Overview of candidate drug molecules for hyperphagia in PWS*

Drug (company)	Mechanism of action	Clinical findings	Current status
Livoletide (Millendo Therapeutics)	Unacylated ghrelin analog; designed to alter acylated: unacylated ghrelin ratio	• Phase 2 (47 participants): participants receiving livoletide experienced reduced hyperphagia and food-related behaviours. Phase 2b/3 (> 100 participants): terminated in 2020 due to a lack of clinically significant benefit [58, 59]	Discontinued
Beloranib (Zafgen, Inc.)	MetAP2 inhibitor (initially developed for cancer); promotes fat oxidation and decreases lipogenesis	• Phase 3(107 participants): participants receiving beloranib experienced weight loss and decreased hyperphagia scores. However, the trial was stopped early due to serious thromboembolic events (two fatal PEs, two DVTs) and psychiatric AEs (psychosis) [60]	Discontinued
Carbetocin (Levo Therapeutics, Acadia Pharmaceuticals)	Synthetic analogue of oxytocin; thought to promote fat loss while preserving muscle and bone mass	• Phase 3 (130 participants aged 7–18 years): participants who received the 3.2 mg dose experienced nominally significant improvements in HQ-CT, PADQ, and CGI-C scores, with sustained effects in long-term follow-up [15] • Global phase 3 (170 participants aged 5–30 years): to evaluate the 3.2 mg three-times-daily dose [61]. Post-meeting update: Carbetocin was discontinued based on the trial failing to meet its primary endpoint (i.e., change from baseline to Week 12 on the HQ-CT) [62]	Discontinued
ARD-101 (Aardvark Therapeutics)	Activate bitter taste receptors in the gut, stimulating the release of satiety-related gut hormones such as GLP-1, GLP-2, and CCK	• Phase 2 (12 participants): 11 participants treated with ARD-101 for 28 days experienced decreased hyperphagia, with mean 7-point reduction in HQ-CT scores, and four experienced near-complete resolution of hunger; delayed gastric emptying but increased motility in the lower gut; generally well tolerated, with manageable side effects [63] • Phase 3 (90 participants): Higher doses of ARD-101 were being investigated in a 12-week, placebo-controlled trial [64]. The trial was paused in February 2026 based on cardiac observations in a separate healthy volunteer study [65]	Development paused
DCCR (Soleno Therapeutics, a Neurocrine Biosciences company)	Potent KATP channel agonist that crosses the blood–brain barrier and acts on both gut and brain	• Phase 3 (> 120 participants): Participants treated with DCCR experienced reduced hyperphagia, improved metabolic health, improved body composition; and improved behavioural symptoms; generally well tolerated, with manageable side effects [66–69] • Safety profile is generally consistent with known effects of diazoxide; most common AEs: hypertrichosis, peripheral oedema, and hyperglycaemia [66]	FDA approved (March 2025)
Setmelanotide (Rhythm Pharmaceuticals)	MC4R agonist	• Phase 2: in interim analysis, participants receiving setmelanotide experienced reduced BMI and hyperphagia [70] • Phase 3: pending	In development

^a^Status as of March 2, 2026

AE, adverse event; CCK, cholecystokinin; DCCR, diazoxide choline extended-release; *DVT*,* deep vein thrombosis; GLP*,* glucagon-like peptide;* MetAP2, methionine aminopeptidase 2; MC4R, melanocortin 4 receptor; PE, pulmonary embolism; POMC, pro-opiomelanocortin; PWS, Prader-Willi syndrome

Diazoxide choline extended-release (DCCR), a KATP channel agonist, represents a significant advance in the treatment of hyperphagia in PWS. While the phase 3 DESTINY PWS trial did not meet its primary endpoint in the overall study population, improvements were observed across several clinically relevant measures, and a subsequent randomised withdrawal study demonstrated the benefit of continued treatment compared with placebo withdrawal. Collectively, these data supported FDA approval of DCCR (VYKAT XR^®^) in March 2025 for the treatment of hyperphagia in adults and children aged 4 years and older with PWS [66–69, 71]. Participants noted that pharmacological therapies may reduce symptoms burden and complement existing management strategies; however, they should be integrated with, rather than replace, behavioural and environmental approaches.

Other therapies targeting melanocortin pathways and gut-brain signalling are under investigation. Setmelanotide is approved for certain rare genetic obesity disorders, but it is not currently approved specifically for PWS. Preclinical evidence in Magel2-null mice suggests potential relevance of melanocortin 4 receptor agonism in PWS-related appetite and metabolic dysregulation; however, these findings remain preclinical and melanocortin pathway therapies should therefore be described as investigational in PWS [64, 70, 72]. 

Interest in GLP-1 receptor agonists has increased because of their established role in type 2 diabetes and obesity management. However, evidence supporting a direct effect on hyperphagia in PWS remains limited. The randomised paediatric liraglutide study in children and adolescents with PWS and obesity did not meet its co-primary BMI endpoints, and evidence for improvement in hyperphagia was not conclusive. Evidence for semaglutide in PWS remains preliminary and is largely limited to case reports and small observational experiences; therefore, these reports should be interpreted as hypothesis-generating rather than evidence of established efficacy. Overall, GLP-1 receptor agonists may have a role in managing diabetes or obesity in selected individuals with PWS, but they are not currently established treatments for hyperphagia in this population [73–88]. 

Participants also raised concerns regarding gastrointestinal safety and potential risks in the PWS population, particularly given known vulnerabilities in gastrointestinal function, including delayed gastric emptying and chronic constipation. Established gastrointestinal adverse effects of GLP-1 receptor agonists, including nausea, vomiting, diarrhoea, constipation and delayed gastric emptying, are clinically relevant in this context. However, specific evidence regarding gastrointestinal safety in PWS remains limited, and any use in this population should therefore be cautious, individualised and closely monitored. This is consistent with recommendations from the FPWR and IPWSO workshop, which highlighted the need for systematic real-world data collection on GLP-1 receptor agonist efficacy and safety in PWS [89]. 

Long-term effectiveness, safety, patient selection, and optimal integration of pharmacological therapies with behavioural and environmental approaches remain areas of ongoing research.

### Integrating pharmacological and behavioural approaches

While pharmacological therapies represent an important emerging component of management, discussions emphasised that these should be integrated with, rather than replace, behavioural and environmental strategies.

Participants noted that expectations around pharmacological treatments must be carefully managed. While these therapies may reduce appetite drive or behavioural symptoms, they are unlikely to eliminate the need for structured food environments. Over-reliance on medication without appropriate environmental support may lead to suboptimal outcomes.

Conversely, effective pharmacological interventions may, in some individuals, enable greater flexibility in management by reducing the intensity of hyperphagic drive. Whether pharmacotherapy can safely permit gradual relaxation of environmental restrictions, reduce caregiver burden and increase autonomy remains an important question for future research and may represent a meaningful endpoint in future clinical trials.

## Future directions

Discussions highlighted several priorities for future research and clinical practice.

First, there is a need for improved definitions and frameworks for understanding and diagnosing hyperphagia that reflect its multidimensional nature. Second, development of validated multidimensional outcome measures that capture hyperphagic drive, meaningful clinical change, caregiver burden, patient autonomy and functional outcomes is essential.

Identification of objective biomarkers of hyperphagia and evaluation of how pharmacological interventions interact with environmental food security strategies also represent important research priorities. Finally, integration of mechanistic insights with clinical practice may enable more targeted and personalised approaches to treatment.

### Toward integrated mechanistic and clinical models

A consistent theme across discussions was the need to better integrate mechanistic understanding with clinical practice. While advances in neuroimaging, endocrinology and genetics have provided valuable insights, their translation into routine care remains limited.

Participants highlighted the potential for mechanistic stratification of patients based on biological or behavioural profiles, which could inform more targeted interventions. As a potential research framework, individuals with predominant reward-driven behaviours may respond differently to therapies compared with those with more pronounced homeostatic dysregulation.

There was also interest in the application of digital health technologies, including wearable devices and real-time behavioural monitoring, to capture dynamic changes in hyperphagia and provide more granular data on treatment response. Such approaches may help distinguish underlying food-related drive from behavioural manifestations that are influenced by food availability, caregiver supervision and environmental controls.

Ultimately, progress in this field is likely to depend on bridging the gap between mechanistic insight, gained from basic research and real-world clinical experience and patient-related outcomes, with multidisciplinary collaboration playing a central role.

### Recent therapeutic advances in PWS (2025–2026)

The therapeutic landscape for Prader-Willi syndrome (PWS) has advanced considerably since 2024. The FDA approval of VYKAT XR^®^ (diazoxide choline extended-release) in 2025 marked the first approved treatment for hyperphagia in PWS and represents a significant milestone in the field [66–69, 71]. In contrast, development of intranasal carbetocin (ACP-101) was discontinued after the Phase 3 COMPASS PWS study failed to demonstrate a statistically significant improvement in hyperphagia compared with placebo [62]. Despite this setback, research into oxytocin-based therapies continues, with novel candidates such as TNX-2900 under evaluation [90], while interest is also growing in the potential role of GLP-1 receptor agonists, although evidence in PWS remains limited [89]. 

## Conclusions

Hyperphagia in PWS is increasingly understood as a complex feature arising from interactions between hypothalamic dysfunction, neurobehavioural processes and environmental context. Importantly, hyperphagia should be recognised as a neurobiological manifestation of PWS rather than simply excessive eating, with implications for both clinical care and societal understanding of the condition. This perspective helps explain why management requires integrated approaches spanning biological, behavioural and environmental domains.

Insights from multidisciplinary discussions reinforce the importance of linking mechanistic understanding of hypothalamic dysfunction with practical models of care. Structured food security measures and supportive environments remain fundamental components of management, even as new therapeutic options emerge. Emerging pharmacological therapies, including DCCR, may provide important additional benefits but should be integrated with, rather than replace, multidisciplinary behavioural and environmental strategies. While DCCR is approved in the United States for the treatment of hyperphagia in PWS, evidence for GLP-1 receptor agonists and melanocortin pathway therapies in PWS remains more limited and/or investigational, and their regulatory status should be considered within the context of local approvals and evolving evidence.

Continued progress in PWS will rely on a truly integrated approach—one that deepens our understanding of the underlying biology, meaningfully incorporates the lived experience of individuals and their families, and actively engages healthcare teams within well-coordinated systems of care that support patients throughout their lives. As a meeting report, the perspectives presented here reflect multidisciplinary expert discussion and meeting-derived recommendations rather than a formal consensus guideline. The International PWS community remain committed to integrating these elements together, ensuring that these advances are meaningful lasting improvements in quality of life for individuals living with PWS and their families. 

## Data Availability

No datasets were generated or analysed during the current study.

## Abbreviations

AE: Adverse event
BMI: Body mass index
CCK: Cholecystokinin
CGI-C: Clinical Global Impression of Change
DCCR: Diazoxide choline extended-release
DVT: Deep vein thrombosis
FDA: Food and Drug Administration
GLP: Glucagon-like peptide
GLP-1RA: Glucagon-like peptide-1 receptor agonist
HQ: Hyperphagia Questionnaire
HQ-CT: Hyperphagia Questionnaire for Clinical Trials
IPWSO: International Prader-Willi Syndrome Organisation
KATP: ATP-sensitive potassium
MC4R: Melanocortin 4 receptor
MetAP2: Methionine aminopeptidase 2
MRI: Magnetic resonance imaging
NDD: Neurodevelopmental disorder
PADQ: Prader-Willi Syndrome Anxiousness and Distress Behaviors Questionnaire
PC1: Prohormone convertase 1
PE: Pulmonary embolism
POMC: Pro-opiomelanocortin
PWS: Prader-Willi syndrome
PWSA: Prader-Willi Syndrome Association
